# Long-term effectiveness of one and two doses of a killed, bivalent, whole-cell oral cholera vaccine in Haiti: an extended case-control study

**DOI:** 10.1016/S2214-109X(18)30284-5

**Published:** 2018-08-10

**Authors:** Molly F Franke, Ralph Ternier, J Gregory Jerome, Wilfredo R Matias, Jason B Harris, Louise C Ivers

**Affiliations:** a Department of Global Health and Social Medicine, Boston, MA, USA; b Department of Pediatrics, Boston, MA, USA; c Harvard Medical School, Boston, MA, USA; d Partners In Health, Port au Prince, Haiti, MA, USA; e Center for Global Health, Massachusetts General Hospital, Boston, MA, USA; f Department of Medicine, Brigham and Women's Hospital, Boston, MA, USA; g Division of Pediatric Global Health, Massachusetts General Hospital, Boston, MA, USA

## Abstract

**Background:**

No study of long-term protection following killed oral cholera vaccination has been done outside of the historically cholera-endemic areas of south Asia, or has examined protection after a single-dose vaccination regimen. To address this, we examined the duration of protection of the standard two-dose regimen and an incomplete regimen of one dose up to 4 years after vaccination in Haiti.

**Methods:**

In the setting of two-dose vaccination campaigns with a killed, bivalent, whole-cell oral cholera vaccination, we did a case-control study from October, 2012 through November, 2016. Eligible participants were required to be resident in the vaccine catchment area (Artibonite Department or Central Department) where they were recruited at the start of the study; and be eligible for the vaccination campaign (ie, aged ≥12 months, not pregnant, and living in the region at the time of the vaccine campaign). Patients with cholera had a positive stool culture and were recruited from cholera treatment centres. Community controls were matched to people with cholera by age group, time, and neighbourhood. We did adjusted matched regression analyses to calculate vaccine effectiveness and examine heterogeneity in effectiveness over time. The primary outcome was the effectiveness of one and two oral cholera doses as compared with zero doses from 2 months to 48 months after vaccination, measured by self reporting.

**Findings:**

Among 178 people assigned to the case group and 706 people assigned to the control group, we found no evidence that two-dose effectiveness decreased during follow-up. In adjusted analyses, the average cumulative 4 year effectiveness for two doses was 76% (95% CI 59–86). In contrast, single-dose effectiveness decreased over time in a log-linear fashion, with a predicted vaccine effectiveness of 79% at the end of 12 months (95% CI 43–93), which declined to zero before the end of the second year.

**Interpretation:**

In a setting of epidemic and newly endemic cholera in Haiti, single-dose vaccination with killed, bivalent, whole-cell oral cholera vaccination provided short-term protection; however, vaccination with two doses was required for long-term protection, which lasted up to 4 years after vaccination. These results add to the evidence in support of the use of killed, bivalent, whole-cell oral cholera vaccination as part of comprehensive cholera control plans.

**Funding:**

US National Institute of Allergy and Infectious Diseases of the National Institutes of Health and the Bill & Melinda Gates Foundation.

## Introduction

Understanding the effectiveness and duration of protection afforded by oral cholera vaccines is needed to develop effective vaccination programmes. One meta-analysis of killed oral cholera vaccines showed an average protective efficacy of 58% and average protective effectiveness of 73%.^1^ Although efficacy studies suggest a decline in protection 2 years after oral vaccination^1^, few studies have reported long-term two dose-effectiveness, and no long-term prospective study of effectiveness has been done outside of Asia, where cholera has been endemic for centuries. The absence of studies of the duration of oral cholera vaccines protection outside the historically cholera-endemic areas of south Asia necessitates evaluating these effects in other populations where conditions of previous and ongoing exposure to *Vibrio cholerae* might differ.

Cholera was introduced to Haiti in 2010, and since then has become endemic. Use of killed, bivalent, whole-cell oral cholera vaccines (Shantha Biotechnics, Hyderabad, India) were first implemented as part of a comprehensive response to cholera in selected high cholera-incidence communities in rural and urban Haiti in April, 2012.^2^, ^3^ It was in this context that we initially did a case-control study to evaluate the effectiveness of killed, bivalent, whole-cell oral cholera vaccines in the rural campaign catchment area up to 2 years after vaccination.^2^, ^4^ Killed, bivalent, whole-cell cholera vaccines were subsequently deployed in vaccination campaigns in other communities in Haiti with a high cholera-incidence.

In this case-control study, we expand the analysis of our previously described case-control study of oral cholera vaccines effectiveness in Haiti to evaluate the duration of protection of the standard two-dose regimen up to 4 years following vaccination. We also examine the duration of oral cholera vaccines-associated protection after a single dose, which has important implications in the studied setting in which vaccine supply is scarce.

**Research in context**

**Evidence before the study**

A systematic review of PubMed and meta-analysis published in October, 2017 reviewed randomised controlled trials and observational studies that reported estimates of direct protection against medically-attended cholera conferred by killed oral cholera vaccines (bivalent whole-cell vaccine and or whole-cell vaccine with B-subunit). They included seven trials and six observational studies (in South Asia, African countries, and Peru) and found that vaccination with two doses of killed oral cholera vaccines offered moderate to high protection against medically attended cholera for the first 3 years after vaccination. There was some evidence to suggest protection beyond 3 years. With regard to single-dose protection, few studies had this as a primary endpoint, but those that did found statistically significant protection over the short-term (eg, up to 1 year after vaccination, protection was 69% effectiveness [95% CI 35–85] from two observational studies and 40% efficacy from one trial [95% CI 11–60]). There were no studies with single-dose protection as a primary endpoint with follow-up of more than 6 months after vaccination and all data on single-dose protection were from populations with regularly occurring cholera transmission. To supplement this review, we searched PubMed using the search terms “cholera” and “vaccine” and (“efficacy” or “effectiveness” or “protect”) and without language restriction for articles published from July 9, 2016, to Nov 21, 2017 and identified 36 articles, none of which reported direct protection of vaccination with a single dose of killed oral cholera vaccines or long-term protection with two-dose vaccination, disaggregated over time.

**Added value of this study**

This study fills three crucial knowledge gaps related to the long-term effectiveness of vaccination with killed, bivalent, whole-cell oral cholera vaccines. First, this is the first study, to our knowledge, to report the field effectiveness of two doses of killed, bivalent, whole-cell oral cholera vaccines up to 4 years after vaccination. We found consistent protection with two killed, bivalent, whole-cell oral cholera vaccines doses, which remained unchanged throughout the 4 years of follow-up (cumulative, adjusted 4 year effectiveness was 76%). Second, we provide the first evidence of long-term protection with two doses of killed, bivalent, whole-cell oral cholera vaccines outside of south Asia, where cholera is historically endemic. The three existing studies of long-term protection of killed oral cholera vaccines were done in India and Bangladesh, where cholera has been endemic for centuries. We did this study in Haiti, where cholera is newly endemic and where ongoing exposure to cholera might differ from places with historically endemic cholera. Third, we provide the first estimates of the duration of protection with a single dose of killed, bivalent, whole-cell oral cholera vaccines. We found a high prevalence of protection during the first year, which declined to zero by the end of the second year after vaccination. These data are crucial for understanding the optimal use of oral cholera vaccines in the context of comprehensive cholera control and prevention.

**Implications of all the available evidence**

Evidence suggests that although single-dose killed, bivalent, whole-cell oral cholera vaccines campaigns are useful in the short term, two-doses are required for long-term protection. These results add to the evidence in support of the use of killed, bivalent, whole-cell oral cholera vaccines as part of comprehensive cholera control plans and of investment in continued support of a global stockpile of killed, bivalent, whole-cell oral cholera vaccines.

## Methods

### Study design

The catchment area for the present study corresponded to those of two vaccination campaigns. The first campaign targeted two rural communities (Bocozel and Grand Saline) in the Artibonite Department and took place from April 15 to June 19, 2012, and the second campaign took place in Mirebalais in the Central Department from Aug 25 to Sept 19, 2014. Each campaign aimed to deliver two oral doses of the vaccine 14 days apart. Details on study design, setting, and participant recruitment have been previously described.^4^ Participant recruitment began in October, 2012 in the Artibonite Department and November, 2014 in the Central department and occurred continuously through November, 2016. Because the vaccination campaign in the Artibonite Department occurred more than 2 years before the campaign in the Central Department, participants from the Artibonite Department contributed the majority of information regarding effectiveness beyond 24 months.

Eligible participants were required to meet two conditions: residency in the vaccine catchment area where they were recruited at the start of the study and eligiblity for the vaccination campaign (ie, aged ≥12 months, not pregnant, and living in the region at the time of the vaccine campaign).

Patients with acute watery diarrhoea—defined as three or more watery, non-bloody stools in a 24 h period with an onset of 3 days or fewer before presentation—were recruited from cholera treatment facilities in the study catchment area. Participants with a stool sample that was culture positive for *V cholerae* O1 were assigned to the cholera group. Only one person per household was enrolled in the study.

For each person with cholera, four community-based people were recruited from their residences and assigned to the control group. People in the community control group were individuals who could be matched to a case by location of residence, enrolment time (within 2 weeks of the case), and age group (1–4 years, 5–15 years, and >15 years) and did not seek treatment for diarrhoea between the first day of participant enrolment in their catchment area and the date of onset of symptoms in their corresponding case. When more than one eligible control was available in a household, an individual of the same sex was selected when possible. If more than one eligible control was available but they were both of different sex to the case, the one most closely matching the person with cholera in age was chosen. In rural Haiti, households are often grouped in a cluster of multigenerational families called lakou.^5^ In choosing controls, study workers approached the home nearest to the person in the patient assigned to the case group's home, excluding homes within the same lakou because we anticipated that exposure to the cholera vaccine was likely to be highly correlated within the lakou. Study workers then approached the next closest residence until four matched controls were enrolled.

### Procedures

Stool samples were collected in sterile containers, and transported in Cary–Blair media to the Haitian National Public Health Laboratory in Port-au-Prince or the Enteric Diseases Laboratory in Saint Marc for subsequent culture on thiosulphate–citrate–bile salts–sucrose agar. Identification of *V cholerae* serogroup O1 at the serotype level was done using a standard slide agglutination method.^6^ PCR was not routinely available in Haiti for cholera diagnosis during the study period.

To collect data for sociodemographic characteristics, cholera risk factors, and self-reported vaccination, study workers interviewed cholera cases at the cholera treatment facility. Within 2 weeks of enrolment, study workers visited patients in their homes to collect additional information on household water storage receptacles and to request vaccination cards for verification, if applicable. Community controls were recruited from and interviewed at their homes. For children and other participants who were unable to respond to interview questions, guardians or a family member proxy responded to questions on behalf of the participant. A study worker abstracted clinical data from the medical charts of cholera cases.

### Assessment of vaccination

Oral cholera vaccination was assessed by self-report during the face-to-face interview. Study workers described the vaccine to study participants in terms of its function, timing of delivery, and mode of administration to differentiate it from other vaccines. If the participant reported receiving the oral cholera vaccines, they were asked how many doses they received. We attempted to verify self-reported vaccination by asking individuals who reported receipt of at least one dose of the vaccine to produce their oral cholera vaccination card during the home visit; only 35% of individuals who reported vaccination were able to produce a vaccination card. This result is probably due to the long time (up to 4 years for some participants) since the vaccination campaigns.Additionally, from 2012 to 2014, digital vaccine registers were reviewed in addition to vaccination cards to confirm vaccination status. From 2014 onwards, the campaigns led by the Ministry of Health used paper registries that were not amenable to review by hand. Because self-reported vaccine prevalence more closely approximated known population vaccine coverage estimates,^2^ we used this as our primary exposure assessment. We conducted sensitivity analyses in which we (1) considered only verified vaccination status as recorded by cards or registries and (2) prioritised verified information over self-report, but used self-reported information if the patient was unable to produce a vaccination card. The latter approach also has been used in other oral cholera vaccines effectiveness case control studies.^7^, ^8^, ^9^

### Statistical analysis

Because there was a delay between the vaccination campaign and study initiation in each catchment area, we did not have data for vaccine effectiveness during the first 2 months following vaccination. Enrolment in the case control study started 2 months following vaccination. The primary outcome was therefore the effectiveness of one and two oral cholera vaccine doses as compared with zero doses from 2 months to 48 months after vaccination. We used indicator variables to model the number of vaccine doses received (one or two) relative to the reference group of no vaccination. We examined whether vaccine effectiveness changed over time by creating interaction terms for the number of doses received and time between vaccination campaign and cholera diagnosis. Time between vaccination campaign and cholera diagnosis was calculated as the time from the date of vaccination to the date of admission to the cholera treatment centre or interview (if the admission date was missing). The date of each participant's vaccination was established on the basis of the midpoint of the vaccination campaign dates for each department (May 17, 2012 for Artibonite Department and Sept 6, 2014 for the Central Department) and the location of each participant's residence. We modelled time since vaccination (up to 48 months after vaccination) as a linear variable and as a natural cubic spline with three equally spaced knots. We compared models with the linear form to those with a spline using the Akaike information criterion. We used Cochran's Q test to test for homogeneity in vaccine effectiveness estimates across the two study sites.

We calculated odds ratios, 95% CIs, and p values using conditional logistic regression, which accounted for matching factors. In multivariable analyses, we additionally adjusted for the following cholera risk factors, identified a priori, that were found to be associated with cholera (at p<0·20) in univariable analyses: female sex, respondent was participant, ever attended school, main toilet is latrine, reports knowing how to treat water, reports always treating water, household buys water, water source (ie, from pump, treated water, bottled water, rain water, and well), water treatment method (ie, tablets, boiling, and chlorine), same water source used for washing and drinking, makes a living by agriculture, makes a living by fishing, consumed food or beverage outside of home in the last week, ate raw fruits or vegetables in the last week, knowledge on how to avoid cholera (ie, heating food, not going to the bathroom near water source, and another way not included in list), hand washing habits (ie, before and after touching a baby, and at another time not included in list), member of household had diarrhoea in the last 7 days, water vessel cover (ie, uncovered, covered, and partially covered), water vessel has a tap, size of opening on water vessel (narrow versus wide), and more than 29 min (75th percentile) on foot from home to river. This approach was used to narrow a list of biologically plausible cholera risk factors while still allowing control for the most likely confounders in this dataset. Because we matched broadly by age category, we included age as a continuous variable in multivariable models to adjust for residual confounding by age within an age category.

Data completeness was high for covariate data. Therefore, primary analyses were restricted to cases and controls that had complete information on all covariates. We did sensitivity analyses in which we altered the presumed dates of vaccination (ie, used the beginning and end dates of the vaccination campaign, rather than the midpoint); imputed missing covariate data; and excluded children younger than 5 years of age since vaccine effectiveness is known to be lower in this group. Multiple imputation (n=25) was done using covariate and outcome data and Markov Chain Monte Carlo methods (SAS MI Procedure), and effect estimates were pooled across datasets. Owing to small numbers, we did not do adjusted analyses or examine effectiveness over time in the subgroup of children less than 5 years of age. We calculated vaccine effectiveness using the formula (1-relative risk).^10^

### Bias-indicator analysis

Bias-indicator case-control studies are often conducted in parallel with vaccine effectiveness case-control studies to assess the likelihood of bias in the latter.^4^, ^7^, ^11^, ^12^, ^13^ We previously reported results from the bias-indicator study that we did in tandem with this case-control study. Some sensitivity analyses revealed a bias-indicator estimate that was significantly different from zero; however, we derived similar vaccine effectiveness estimates using both community-based and test-negative controls, which led us to conclude that bias, if present, was minimal.^14^

### Ethical considerations

Written informed consent was obtained for all participants, or from a health-care proxy if the participant was unable to consent. Consent from a parent or guardian was obtained for children younger than 18 years of age, and assent was sought from children aged 7–17 years. The study protocol was approved by Partners Human Research Committee and the Haiti National Bioethics Committee.

Role of the funding sourceThe study funders had no role in the design of the study; collection, analysis, and interpretation of data; in the writing of the report; or in the decision to submit the paper for publication.

## Results

**Figure 1:**
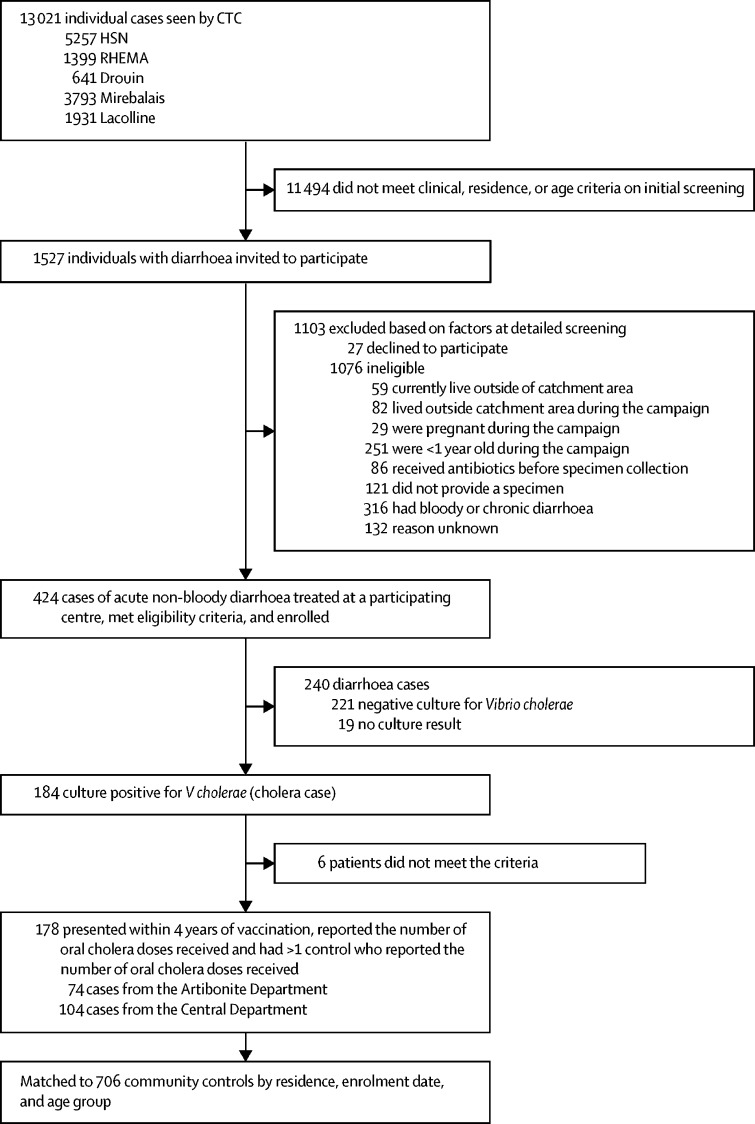
**Overview of enrolment of cholera cases and community controls** CTC=Cholera treatment centre. HSN=Hospital St Nicholas. RHEMA=J Peter Gruits Medical Center.

178 people assigned to the case group and 706 people assigned to the control group were included for analysis (figure 1). 21 of the 177 cases (12%) occurred in children less than 5 years of age. We excluded four people assigned to the cases of cholera group occurring beyond 48 months of follow-up and four participants (two cases of cholera and two controls) for whom we had no information on self-reported number of vaccine doses received (figure 1). Table 1 shows vaccination frequency in cholera cases and controls, stratified by case time from vaccination to cholera diagnosis. 32 cases (18%) occurred more than 2 years after vaccination, with the majority of these (n=23, 72%) occurring in the fourth year, and only nine (28%) cases occurring in the third year after vaccination. Relevant data for sociodemographic characteristics and cholera risk factors for this cohort have been previously reported and are therefore not repeated here.^14^

**Table 1:** Enrolment of cases and controls, by time since vaccination campaign

		**Overall**		**Artibonite Department**		**Central Department**	
		Cases (n=178)	Controls (n=706)	Cases (n=74)	Controls (n=291)	Cases (n=104)	Controls (n=415)
0–12 months from vaccination campaign		54	215	11	44	43	171
	None	28 (52%)	70 (33%)	3 (27%)	2 (5%)	25 (58%)	68 (40%)
	One dose	4 (7%)	23 (11%)	1 (9%)	8 (18%)	3 (7%)	15 (9%)
	Two doses	22 (41%)	122 (57%)	7 (64%)	34 (77%)	15 (35%)	88 (51%)
12–24 months from vaccination campaign		92	367	34	135	58	232
	None	44 (48%)	114 (31%)	10 (29%)	18 (13%)	34 (59%)	96 (41%)
	One dose	15 (16%)	51 (14%)	2 (6%)	11 (8%)	13 (22%)	40 (17%)
	Two doses	33 (36%)	202 (55%)	22 (65%)	106 (79%)	11 (19%)	96 (41%)
24–36 months from vaccination campaign		9	36	6	24	3	12
	None	1 (11%)	17 (47%)	1 (17%)	8 (33%)	0 (0%)	9 (75%)
	One dose	1 (11%)	0 (0%)	0 (0%)	0 (0%)	1 (33%)	0 (0%)
	Two doses	7 (78%)	19 (53%)	5 (83%)	16 (67%)	2 (67%)	3 (25%)
36–48 months from vaccination campaign		23	88	23	88	0	0
	None	12 (52%)	13 (15%)	12 (52%)	13 (15%)	..	..
	One dose	5 (22%)	2 (2%)	5 (22%)	2 (2%)	..	..
	Two doses	6 (26%)	73 (83%)	6 (26%)	73 (8%3)	..	..

Two-dose vaccine effectiveness estimates across the 4 year follow-up period were 70% (95% CI 54–80) for unadjusted data and 76% (59–86) for adjusted data (p<0·0001 for both; table 2). Using Akaike information criterion, we concluded that neither the model with a natural cubic spline (predicted vaccine effectiveness estimates by month shown in appendix), nor the model with a linear interaction fit the data better than the model assuming a constant vaccine effectiveness across the 4 years of follow-up (p value for interaction when time since vaccination was modelled as a linear variable=0·57). The Akaike information criterions for the different models are compared in the appendix. We concluded that two-dose effectiveness did not vary importantly throughout the 4 year follow-up period and that any observed variability was due to chance. The interaction term between two-dose vaccination and time since vaccination was therefore excluded from the final model.

**Table 2:** Analyses of two-dose vaccine effectiveness with bivalent whole-cell oral cholera vaccine across 4 years of follow-up

	**Unadjusted**		**Adjusted**	
	VE (95% CI)	p value	VE (95% CI)	p value
**Complete case analyses**				
All ages	70* (54–80)	<0·0001	76† (59–86)	<0·0001
≥5 years of age	73* (58–83)	<0·0001	77† (58–88)	<0·0001
**Multiply imputed analyses**				
All ages	Not applicable	..	76* (59–86)	<0·0001
≥5 years of age	Not applicable	..	76* (58–87)	<0·0001

VE=Vaccine effectiveness.

* Unadjusted analyses and those with multiple imputation included 178 cases and 706 controls when all ages were included, and 157 cases and 623 controls when children <5 years of age were excluded.

† Adjusted complete case analysis of all ages includes 166 cases and 696 controls. Adjusted complete case analysis excluding children <5 years of age, includes 145 cases and 616 controls.

**Figure 2:**
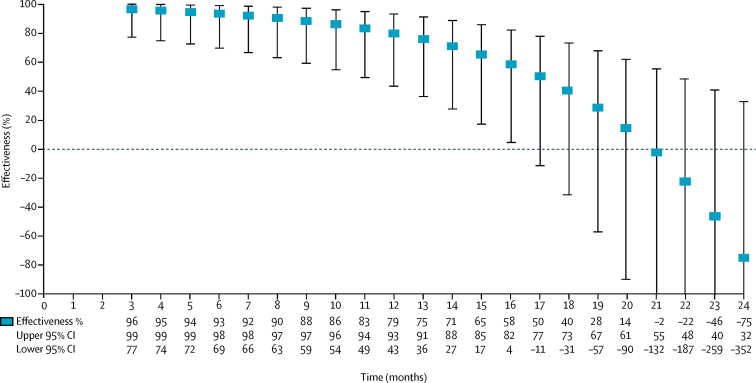
Adjusted estimates of vaccine effectiveness of a single dose of killed, bivalent, whole cell oral cholera vaccines over 24 months

25 cases (14%) and 76 controls (11%) received a single vaccine dose (table 1). Adjusted single-dose effectiveness estimates decreased log-linearly with each month since vaccination (p_interaction_=0·0004; model estimates in appendix), and modelling time since vaccination using a natural cubic spline did not improve model fit relative to the linear form (appendix). We observed high vaccine effectiveness ranging from 96% to 79% during the first year after vaccination, which eventually declined to 0 after month 20, though the lower 95% confidence bound first dropped below 0 after month 16 (figure 2).

The distribution of the cases according to time since vaccination are shown (table 3). Only two (10%) of these 21 cases in children aged less than 5 years received a single dose, whereas 13 (62%) received two doses. Unadjusted one-dose vaccine effectiveness was 10% in this group (95% CI −468 to 86), and two-dose vaccine effectiveness was 32% (95% CI –117 to 79). When we restricted analyses to the first 2 years of follow-up, unadjusted one dose was 67% (95% CI –250 to 97) and unadjusted two dose effectiveness was 48% (95% CI −75 to 85). None of these estimates was significant. When we examined vaccine effectiveness in children who were younger than 5 years of age at the time of vaccination (vs at the time of cholera), there were no notable changes (appendix).

**Table 3:** Cases in children younger than 5 years, by time since vaccination

	**n (%)**
**0–12 months from vaccination (n=53)**	
None	0 (0)
One dose	0 (0)
Two doses	4 (100)
Total	4 (8)*
**12–24 months from vaccination (n=93)**	
None	6 (40)
One dose	1 (7)
Two doses	8 (53)
Total	15 (16)*
**24–36 months from vaccination (n=9)**	
None	0 (0)
One dose	1 (50)
Two doses	1 (50)
Total	2 (22)*
**36–48 months from vaccination (n=22)**	
Total	0 (0)

* Percentage of all cases during that time period that occurred in children <5 years of age.

Results were similar for both one dose and two dose analyses when we excluded children younger than 5 years of age (table 2, appendix), when we did multivariable analyses on multiply imputed datasets to account for the small amount of missing covariate data (table 2, appendix) and when we varied the presumed vaccination dates (appendix). We found no evidence of heterogeneity of effect across the two study sites (appendix) and model estimates were similar when we restricted data to the first two years of follow-up (appendix). Average two dose effectiveness was consistent regardless of whether vaccination was assessed by self-report, verification, or a combination of the two methods (appendix). In contrast, single dose effectiveness was similar to primary analyses when we accepted self-report for individuals who could not produce a vaccination card, but was not significantly different from zero when we considered a participant vaccinated only if it could be verified by card or registry (appendix).

## Discussion

We found consistent, lasting protection of 76% with two doses of killed, bivalent, whole-cell oral cholera vaccines up to 4 years after vaccination. These data from Haiti are the first, to our knowledge, from outside of Asia to provide evidence for the long-term effectiveness of two doses of killed, bivalent, whole-cell oral cholera vaccines and support the role of vaccination as part of cholera control efforts. Our findings are consistent with the two-dose long-term vaccine protection of killed, bivalent, whole-cell oral cholera vaccines reported in studies done in India (65% and 69%)^15^, ^16^ and Vietnam (50%). Although the long-term effectiveness estimate was lower in the Vietnam study, nearly a third of cholera cases lacked culture confirmation; if some of these clinically diagnosed cases did not have cholera this would have been expected to attenuate vaccine effectiveness estimates. The two-dose vaccine effectiveness we observed in children under 5 years of age, although not significant, corresponds to the average estimates reported in a recent meta-analysis^1^ (30% [95% CI 15–42]) and highlights the need for alternative strategies to reduce cholera incidence in this vulnerable group.^13^

Cholera vaccination with the standard two-dose regimen can be challenging, especially in settings of crisis, conflict, or humanitarian emergency. Because of this, the use of a single dose has been proposed as a temporising measure to reduce cholera risk in the short term, leading to an interest in understanding the protective effect of a single dose of oral cholera vaccines.^17^, ^18^ One modelling study suggested that the one-dose approach could avert more cholera cases by generating greater herd immunity relative to what could be achieved by vaccinating fewer people with two doses; however, these findings were based on scarce short-term single-dose efficacy data.^19^ Our estimates of single-dose effectiveness appeared higher or extended, or both, relative to those previously reported; however, confidence intervals around our estimates were wide owing to a relatively small number of individuals who received a single dose (ie, we aimed to give everyone in the campaign two doses). The only single-dose efficacy trial reported 40% (95% CI 11–60) protection over 6 months,^20^ whereas a case-cohort analysis reported 87% (70–100) effectiveness within 2 months.^21^ We estimated effectiveness at 96% (95% CI 77–99) at 3 months and 93% (69–98) at 6 months. Similarly, although the average of two studies reporting one-dose cumulative effectiveness was 69% (95% CI 35–85) over the course of a year,^1^ we found an effectiveness of 79% (43–93) at the end of that year. Importantly, our analysis allowed us to estimate effectiveness at a given point in time, versus providing average estimates over an interval during which effectiveness might be declining; therefore, our estimates are not directly comparable with the averaged estimates. The benefit of this analytic approach is that it allowed us to examine changes in effectiveness over time. For vaccination with a single, killed, bivalent, whole-cell oral cholera vaccines dose, we found that effectiveness appeared to diminish completely within 2 years; however, confidence intervals included 0% effectiveness after 16 months. Future studies with larger numbers of single-dose recipients will allow more precise estimates of short-term and long-term protection, and additional work is needed to understand the contexts and implementation strategies in which single-dose vaccination is most appropriate and effective.

The limitations of this study include the relatively small number of people vaccinated with a single dose and the relatively small number of cholera cases occurring at 3 years or more after vaccination. These small numbers resulted in wider confidence intervals around single dose effectiveness estimates and could have concealed small declines in two-dose effectiveness over time. Furthermore, we lacked a gold-standard vaccination registry for vaccination assessment. Self-report of the number of vaccine doses received is imperfect as it might be differentially recalled by cases and controls. Relying on vaccination cards for vaccination assessment is also problematic because individuals could misplace their cards. This factor was a common occurrence in the present study: documented vaccine uptake in the Artibonite Department was between 79% and 92% in Bocozel and 63% in Grand Saline; however, only 44% of controls in this region could produce a vaccination card.^2^ This could be due to the environmental conditions in which it is difficult to keep paper cards safely stored, or due to a shortage of experience of vaccination cards for adults in the region, or both. This limitation did not appear to affect long-term two dose oral cholera vaccines estimates, which were robust to vaccine assessment method. However, when we calculated one-dose effectiveness using verified vaccination, we were left with only 20% of our initial sample size of participants who reported receiving one dose, and the protective association of a single dose was no longer evident. Although this latter analysis is highly prone to bias, our estimates of the duration of protection associated with a single dose must nonetheless be interpreted with caution.

A third limitation is that we approximated vaccination date on the basis of area of residence at the time of study recruitment and the midpoint date of the vaccination campaign in that catchment area. Given that campaign dates for each catchment area did not span more than 65 days and that results were unchanged in sensitivity analyses using the start and end dates for each campaign, we do not believe this limitation affected our study findings. PCR testing for cholera was not available in Haiti at the time of our study and we relied on stool culture. However, we do not expect the absence of PCR for cholera diagnosis to have affected our results because the consequence of a less sensitive diagnostic would have been the misclassification and subsequent exclusion of cholera cases with a false negative culture result and this is unlikely to be associated with cholera vaccination.

In conclusion, vaccination with two doses of killed, bivalent, whole-cell oral cholera vaccines provided consistent protection against medically attended cholera over 4 years in Haiti, where cholera has recently become endemic. Furthermore, single-dose vaccination offered short-term protection against medically attended cholera. Our findings are generalisable to other settings with epidemic and newly endemic cholera. These results add to the evidence in support of the use of killed, bivalent, whole-cell oral cholera vaccines as part of comprehensive cholera control plans and add evidence to the investment case for continued support of a global stockpile of killed, bivalent, whole-cell oral cholera vaccines.

## References

[1] Bi Q, Ferreras E, Pezzoli L (2017). Protection against cholera from killed whole-cell oral cholera vaccines: a systematic review and meta-analysis. Lancet Infect Dis.

[2] Ivers LC, Teng JE, Lascher J (2013). Use of oral cholera vaccine in Haiti: a rural demonstration project. Am J Trop Med Hyg.

[3] Juste MAJ, Francois J, Peck M (2013). Cholera vaccination in urban Haiti. Am J Trop Med Hyg.

[4] Ivers LC, Hilaire IJ, Teng JE (2015). Effectiveness of reactive oral cholera vaccination in rural Haiti: a case-control study and bias-indicator analysis. Lancet Glob Health.

[5] Edmond YM, Randolph SM, Richard GL (2007). The lakou system: a cultural, ecological analysis of mothering in rural Haiti. J Pan Afr Stud.

[6] WHO (2003). Manual for the laboratory identification and antimicrobial susceptibility testing of bacterial pathogens of public health concern in the developing world. http://www.who.int/csr/resources/publications/drugresist/WHO_CDS_CSR_RMD_2003_6/en/.

[7] Luquero FJ, Grout L, Ciglenecki I (2014). Use of *Vibrio cholerae* vaccine in an outbreak in Guinea. N Engl J Med.

[8] Anh DD, Lopez AL, Thiem VD (2011). Use of oral cholera vaccines in an outbreak in Vietnam: a case control study. PLoS Negl Trop Dis.

[9] Ferreras E, Chizema-Kawesha E, Blake A (2018). Single-dose cholera vaccine in response to an outbreak in Zambia. N Engl J Med.

[10] Halloran ME, Struchiner CJ, Longini IM (1997). Study designs for evaluating different efficacy and effectiveness aspects of vaccines. Am J Epidemiol.

[11] Shapiro ED (2004). Case-control studies of the effectiveness of vaccines: validity and assessment of potential bias. Pediatr Infect Dis J.

[12] Lucas MES, Deen JL, von Seidlein L (2005). Effectiveness of mass oral cholera vaccination in Beira, Mozambique. N Engl J Med.

[13] Thiem VD, Deen JL, von Seidlein L (2006). Long-term effectiveness against cholera of oral killed whole-cell vaccine produced in Vietnam. Vaccine.

[14] Franke MF, Jerome JG, Matias WR (2017). Comparison of two control groups for estimation of oral cholera vaccine effectiveness using a case-control study design. Vaccine.

[15] Wierzba TF, Kar SK, Mogasale VV (2015). Effectiveness of an oral cholera vaccine campaign to prevent clinically-significant cholera in Odisha State, India. Vaccine.

[16] Bhattacharya SK, Sur D, Ali M (2013). 5 year efficacy of a bivalent killed whole-cell oral cholera vaccine in Kolkata, India: a cluster-randomised, double-blind, placebo-controlled trial. Lancet Infect Dis.

[17] Parker LA, Rumunu J, Jamet C (2017). Neighborhood-targeted and case-triggered use of a single dose of oral cholera vaccine in an urban setting: feasibility and vaccine coverage. PLoS Negl Trop Dis.

[18] Parker LA, Rumunu J, Jamet C (2017). Adapting to the global shortage of cholera vaccines: targeted single dose cholera vaccine in response to an outbreak in South Sudan. Lancet Infect Dis.

[19] Azman AS, Luquero FJ, Ciglenecki I, Grais RF, Sack DA, Lessler J (2015). The impact of a one-dose versus two-dose oral cholera vaccine regimen in outbreak settings: a modeling study. PLoS Med.

[20] Qadri F, Wierzba TF, Ali M (2016). Efficacy of a single-dose, inactivated oral cholera vaccine in Bangladesh. N Engl J Med.

[21] Azman AS, Parker LA, Rumunu J (2016). Effectiveness of one dose of oral cholera vaccine in response to an outbreak: a case-cohort study. Lancet Glob Health.

