# Supplementary material for: Long-term effectiveness of one and two doses of a killed, bivalent, whole-cell oral cholera vaccine in Haiti: an extended case-control study
Source: Lancet Glob Health. 2018 Aug 10;6(9):e1028–35. doi: 10.1016/S2214-109X(18)30284-5 (PMC6190920; doi:10.1016/S2214-109X(18)30284-5)
Supplement: Supplementary appendix [file mmc1.pdf]

THE LANCET
Global Health

## Supplementary appendix

This appendix formed part of the original submission and has been peer reviewed. We post it as supplied by the authors.

Supplement to: Franke MF, Ternier R, Jerome JG, Matias WR, Harris JB, Ivers LC. Long-term effectiveness of one and two doses of a killed, bivalent, whole-cell oral cholera vaccine in Haiti: an extended case-control study. *Lancet Glob Health* 2018; **6:** e1028–35.

# Web Extra Material

**Appendix Table 1. AIC values for conditional logistic regression models with different approaches to modeling the interaction between number of vaccine doses and time since vaccination.**

| Model | Two doses | | One dose | | AIC |
|---|---|---|---|---|---|
| | Interaction-term included? | If interaction-term included, how was time modeled? | Interaction-term included? | If interaction-term included, how was time modeled? | |
| 1 | No | -- | Yes | Linear | 411.882 |
| 2 | Yes | Natural cubic spline, three equally space knots | Yes | Linear | 412.270 |
| 3 | Yes | Linear | Yes | Linear | 413.553 |
| 4 | No | -- | Yes | Natural cubic spline, three equally space knots | 413.634 |
| 10 | No | -- | No | -- | 426.401 |

**Appendix Table 2. Adjusted model estimates for single dose vaccine effectiveness and interaction with time since vaccination**

| | Unadjusted | | | Adjusted[a,b,c] | | | Adjusted, multiple imputation[a] | | |
|---|---|---|---|---|---|---|---|---|---|
| | OR | 95% CI | p-value | OR | 95% CI | p-value | OR | 95% CI | p-value |
| All ages (178 cases, 706 controls) | | | | | | | | | |
| One dose | 0·11 | [0·03, 0·45] | 0·002 | 0·02 | [0·00, 0·18] | 0·0003 | 0·04 | [0·01, 0·25] | 0·0006 |
| Interaction between one dose and time since diagnosis (in months, linear) | 1·12 | [1·04, 1·20] | 0·003 | 1·20 | [1·08, 1·32] | 0·0004 | 1·17 | [1·07, 1·28] | 0·001 |
| Excluding children <5 (157 cases, 623 controls) | | | | | | | | | |
| One dose | 0·13 | [0·03, 0·55] | 0·005 | 0·03 | [0·00, 0·30] | 0·002 | 0·06 | [0·01, 0·43] | 0·005 |
| Interaction between one dose and time since diagnosis (in months, linear) | 1·10 | [1·03, 1·18] | 0·007 | 1·17 | [1·05, 1·30] | 0·004 | 1·14 | [1·03, 1·26] | 0·01 |

[a] Model adjusted for matching factors and female sex, age (continuous), respondent was self, ever attended school, main toilet is latrine, reports knowing how to treat water, reports always treating water, household buys water, water source (from pump, treated water, bottled water, rain water, well), water treatment method (tablets, boiling, chlorine), same water source used for washing and drinking, makes a living by agriculture, makes a living by fishing, consumed food or beverage outside of home in the last week, ate raw fruits or vegetables in the last week, knowledge on how to avoid cholera (heating food, not going to the bathroom near water source, "other" way not included in list), hand washing habits (before and after touching a baby, at "other" time not included in list), member of household had diarrhea in last 7 days, water vessel cover (uncovered, covered, partially covered), water vessel has a tap, size of opening on water vessel (narrow versus wide) and more than 29 minutes (75$^{th}$ percentile) on foot from home to river

[b] Adjusted analyses of all ages includes 166 cases and 696 controls

[c] Adjusted analysis excluding children less than 5 years includes 145 cases and 616 controls

**Appendix Figure 1. Adjusted estimates of vaccine effectiveness of two-doses of BiWCV over 48-months**

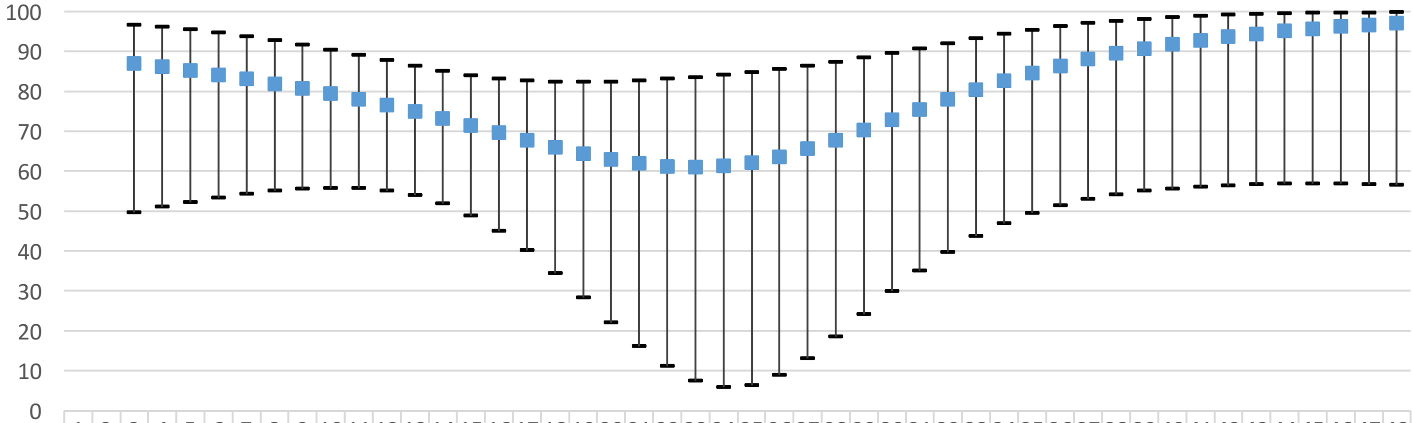


| | 1 | 2 | 3 | 4 | 5 | 6 | 7 | 8 | 9 | 10 | 11 | 12 | 13 | 14 | 15 | 16 | 17 | 18 | 19 | 20 | 21 | 22 | 23 | 24 |
|---|---|---|---|---|---|---|---|---|---|---|---|---|---|---|---|---|---|---|---|---|---|---|---|---|
| Effectiveness% | | | 87 | 86 | 85 | 84 | 83 | 82 | 81 | 79 | 78 | 77 | 75 | 73 | 72 | 70 | 68 | 66 | 64 | 63 | 62 | 61 | 61 | 61 |
| Lower 95% CI | | | 50 | 51 | 52 | 53 | 54 | 55 | 56 | 56 | 56 | 55 | 54 | 52 | 49 | 45 | 40 | 34 | 28 | 22 | 16 | 11 | 8 | 6 |
| Upper 95% CI | | | 97 | 96 | 95 | 95 | 94 | 93 | 92 | 90 | 89 | 88 | 86 | 85 | 84 | 83 | 83 | 82 | 82 | 82 | 83 | 83 | 84 | 84 |

| | 25 | 26 | 27 | 28 | 29 | 30 | 31 | 32 | 33 | 34 | 35 | 36 | 37 | 38 | 39 | 40 | 41 | 42 | 43 | 44 | 45 | 46 | 47 | 48 |
|---|---|---|---|---|---|---|---|---|---|---|---|---|---|---|---|---|---|---|---|---|---|---|---|---|
| Effectiveness% | 62 | 64 | 66 | 68 | 70 | 73 | 76 | 78 | 80 | 83 | 85 | 86 | 88 | 90 | 91 | 92 | 93 | 94 | 94 | 95 | 96 | 96 | 97 | 97 |
| Lower 95% CI | 6 | 9 | 13 | 19 | 24 | 30 | 35 | 40 | 44 | 47 | 49 | 51 | 53 | 54 | 55 | 56 | 56 | 56 | 57 | 57 | 57 | 57 | 57 | 57 |
| Upper 95% CI | 85 | 86 | 86 | 87 | 88 | 90 | 91 | 92 | 93 | 94 | 95 | 96 | 97 | 98 | 98 | 99 | 99 | 99 | 99 | 99 | 10 | 10 | 10 | 10 |

This figure shows vaccine effectiveness by time since vaccination, which was modeled using natural cubic splines with three degrees and three equally-spaced internal knots. Using Akaike information criteria, we concluded that this model did not fit the data better than one that assumes a constant vaccine effectiveness across the four years of follow-up.

**Appendix Figure 2. Adjusted estimates of vaccine effectiveness of a single dose of BiWCV OCV over 24-months, excluding children less than 5 years of age**

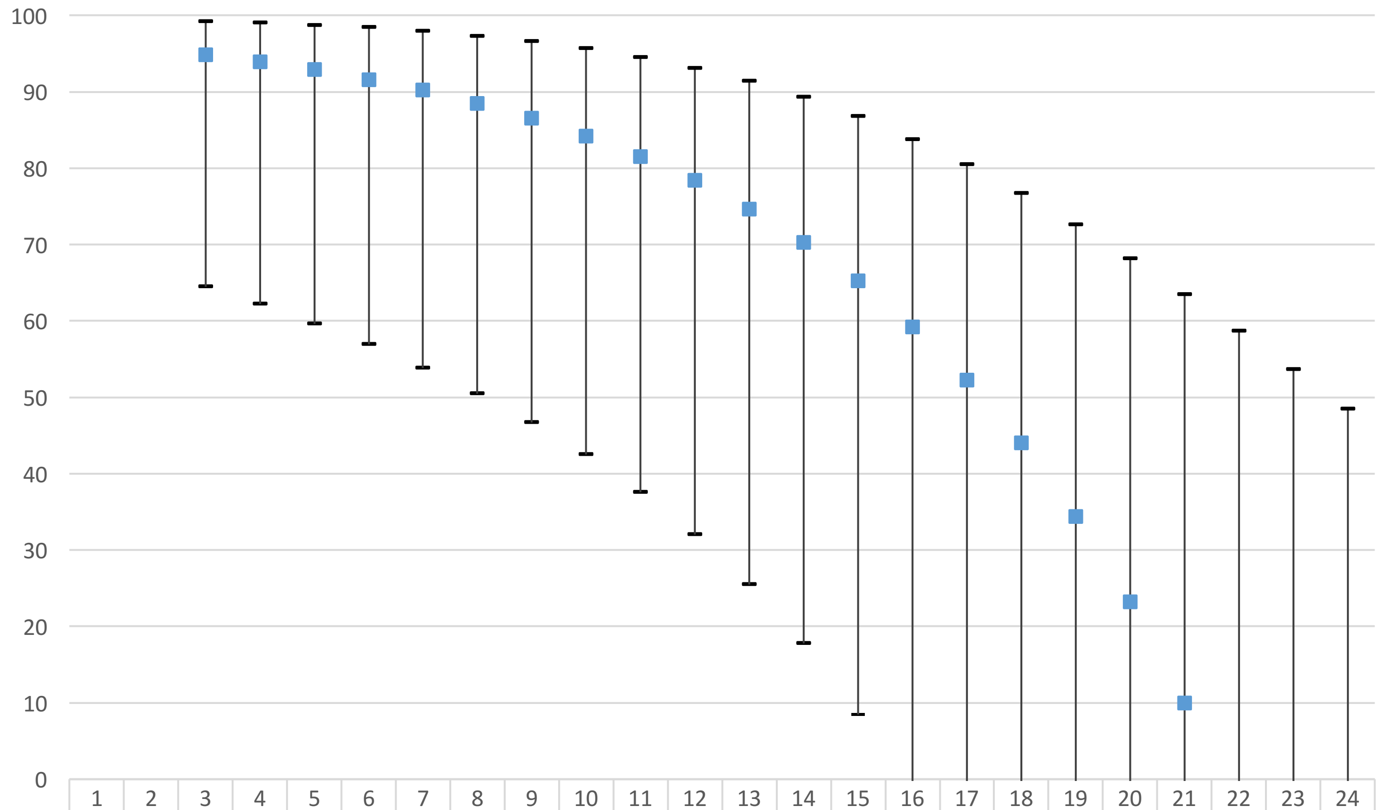


| | 1 | 2 | 3 | 4 | 5 | 6 | 7 | 8 | 9 | 10 | 11 | 12 | 13 | 14 | 15 | 16 | 17 | 18 | 19 | 20 | 21 | 22 | 23 | 24 |
|---|---|---|---|---|---|---|---|---|---|---|---|---|---|---|---|---|---|---|---|---|---|---|---|---|
| % Effectiveness | | | 95 | 94 | 93 | 92 | 90 | 89 | 87 | 84 | 82 | 78 | 75 | 70 | 65 | 59 | 52 | 44 | 34 | 23 | 10 | -5 | -24 | -45 |
| Lower 95% CI | | | 65 | 62 | 60 | 57 | 54 | 51 | 47 | 43 | 38 | 32 | 26 | 18 | 9 | -3 | -17 | -35 | -57 | -85 | -122 | -169 | -229 | -307 |
| Upper 95% CI | | | 99 | 99 | 99 | 98 | 98 | 97 | 97 | 96 | 95 | 93 | 91 | 89 | 87 | 84 | 81 | 77 | 73 | 68 | 64 | 59 | 54 | 49 |

**Vaccine effectiveness in children vaccinated at less than five years of age**

Twenty eight of the 178 cases (16%) occurred in children who were vaccinated when younger than five. Table 8 shows the distribution of the 28 cases who were vaccinated at an age of less than five years, stratified by time since vaccination. Only four (14%) received a single dose, while 16 (57%) received two doses. Unadjusted one dose vaccine effectiveness was -69% in this group [95% CI: -786% to 68%], and two dose vaccine effectiveness was 28% [95% CI: -109% to 75%]. When we restricted analyses to the first two years of follow-up, unadjusted one and two dose effectiveness were 18% [95% CI: -447 to 88%] and 48% [95% CI: 84% to -63%], respectively. None of these estimates was statistically significant.

**Appendix Table 3. Cholera cases in children who were less than five years of age when vaccinated, by time since vaccination**

| | n (%) |
|---|---|
| **0-12 months from vaccination (N=53)** | **6 (11)*** |
| None | 0 (0) |
| One dose | 0 (0) |
| Two doses | 6 (100) |
| **12-24 months from vaccination (N=92)** | **18 (20)*** |
| None | 8 (44) |
| One dose | 2 (11) |
| Two doses | 8 (44) |
| **24-36 months from vaccination (N=9)** | **3 (33)*** |
| None | 0 (0) |
| One dose | 1 (33) |
| Two doses | 2 (66) |
| **36-48 months from vaccination (N=23)** | **1 (4)*** |
| None | 0 (0) |
| One dose | 1 (100) |
| Two doses | 0 (0) |

* Percent of all cases during that time period that occurred in children who were less than five years of age at the time of vaccination

**Appendix Table 4: Sensitivity analyses varying presumed vaccination dates.**

| | Adjusted complete case analyses | |
|---|---|---|
| | OR [a] | 95% CI |
| Start date of campaign | | |
| Two doses | 0.24 | [0.14 – 0.42] |
| One dose | 0.02 | [0.0 – 0.18] |
| Interaction between one dose and time since vaccination | 1.19 | [1.08 – 1.31] |
| End date of campaign | | |
| Two doses | 0.26 | [0.15 – 0.44] |
| One dose | 0.03 | [0.0 – 0.21] |
| Interaction between one dose and time since vaccination | 1.19 | [1.08 – 1.31 |
| Midpoint of campaign, incorporating later Artibonite campaign in children <10 years of age* | | |
| Two doses | 0.24 | [0.14 – 0.41] |
| One dose | 0.02 | [0.0 – 0.18] |
| Interaction between one dose and time since vaccination | 1.19 | [1.08 – 1.32] |

*For more information, see Ivers et al. *Use of Oral Cholera Vaccine in Haiti: A Rural Demonstration Project*. AJTMH. 2013.

**Appendix Table 5. Unadjusted β coefficients for one and two vaccine doses, disaggregated by site and time since vaccination.**

| | Artibonite Department β (std) | Central Department β (std) | Overall β (std) | p-value for difference in β by Department |
|---|---|---|---|---|
| 0 – 24 months | | | | |
| Two doses | -1.28 (0.43) | -1.21 (0.30) | -1.17 (0.24) | 0.89 |
| One dose | -2.68 (1.92) | -1.59 (1.26) | -2.13 (1.06) | 0.64 |
| Interaction between one-dose vaccination and time since vaccination (continuous) | 0.08 (0.13) | 0.09 (0.08) | 0.10 (0.06) | 0.97 |
| 24 - 48 months | | | | |
| Two doses | -1.39 (0.48) | | | |
| One dose | 90.84 (135.6) | -- | -- | -- |
| Interaction between one-dose vaccination and time since vaccination (continuous) | -2.12 (3.23) | | | |

NOTE: We ran two different models, one restricted to the first 24 months of follow-up and one restricted to months 24-48. We tested differences in estimates across the two departments using Cochran's Q test for heterogeneity.

NOTE: The unadjusted overall vaccine effectiveness of two doses, across both study sites, during the first two years of follow-up was 69% [95% CI: 50-81].

**Appendix Table 6. Adjusted odds ratios for one and two vaccine doses and interaction during 24 and 48 months of follow-up.**

| | **0 – 24 months<br>Adjusted OR [95% CI]<br>136 cases, 573 controls** | **0 – 48 months<br>Adjusted OR [95% CI]<br>166 cases, 696 controls** |
|---|---|---|
| Two doses | 0.22 [0.12, 0.41] | 0.24 [0.14, 0.41] |
| One dose | 0.02 [0.0, 0.34] | 0.02 [0.0, 0.18] |
| Interaction between one-dose vaccination and time since vaccination (continuous) | 1.22 [1.02, 1.46] | 1.20 [1.08, 1.32] |

**Appendix Table 7. Adjusted odds ratios, by vaccine assessment method, all ages, complete case analyses.**

| Exposure | Cases (N=166) | Controls (N-696) | OR | 95% CI | p-value |
|---|---|---|---|---|---|
| Self-reported [a,b] | | | | | |
| None | 85 (48) | 214 (30) | Reference | | |
| One | 25 (14) | 76 (11) | 0.02 | [0 – 0.18] | 0.0003 |
| Two | 68 (38) | 416 (59) | 0.24 | [0.14 – 0.41] | <0·0001 |
| Interaction | | | 1.20 | [1.08 – 1.32] | 0.0004 |
| Verified if available, otherwise self-report [b] | | | | | |
| None | | | | | |
| One | 85 (48) | 214 (30) | Reference | | |
| Two | 24 (13) | 71 (10) | 0.08 | [0.01 – 0.51] | 0·0082 |
| Interaction | 69 (39) | 421 (60) | 0.23 | [0.14 – 0.40] | <0·0001 |
| | | | 1.14 | [1.03 – 1.25] | 0·0095 |
| Verified only (if a participant could not produce their card they were considered to be unvaccinated); N=883) [a,c] | | | | | |
| None | 147 (83) | 494 (70) | Reference | | |
| One | 3 (2) | 16 (2) | 0.27 | [0.04 – 1.81] | 0.18 |
| Two | 27 (15) | 196 (28) | 0.23 | [0.12 – 0.47] | <0·0001 |
| Verified only (those who reported vaccination but could not produce their card were excluded)[d] | | | | | |
| None | 85 (74) | 214 (50) | Reference | | |
| One | 3 (3) | 16 (4) | 0.25 | [0.03 – 2.43] | 0.23 |
| Two | 27 (23) | 196 (46) | 0.15 | [0.05 – 0.43] | 0.0004 |

[a] Vaccine effectiveness estimates for self-reported and verified vaccination were previously reported for a similar cohort in *Franke MF, et al. "Comparison of two control groups for estimation of oral cholera vaccine effectiveness using a case-control study design." Vaccine. 2017*

[b] Multivariable analyses include 166 cases, 696 controls

[c] Multivariable analyses include 165 cases, 696 controls; interaction term excluded due to lack of statistical significance and a lower AIC in the model without it.

[d] Multivariable analyses include 106 cases, 417 controls; interaction term excluded due to lack of statistical significance and a lower AIC in the model without it.

**Appendix Table 8: OR and VE estimates for vaccination with a single dose, using verified vaccination assessment (if available) and otherwise self-report.**

| Month | OR | 95% Confidence Limits | | VE | 95% Confidence Limits | |
|---|---|---|---|---|---|---|
| 1 | 0.09 | 0.02 | 0.56 | 91% | 44% | 99% |
| 2 | 0.10 | 0.02 | 0.58 | 90% | 42% | 98% |
| 3 | 0.12 | 0.02 | 0.61 | 88% | 39% | 98% |
| 4 | 0.13 | 0.03 | 0.63 | 87% | 37% | 97% |
| 5 | 0.15 | 0.04 | 0.66 | 85% | 34% | 97% |
| 6 | 0.17 | 0.04 | 0.69 | 83% | 31% | 96% |
| 7 | 0.20 | 0.05 | 0.72 | 81% | 28% | 95% |
| 8 | 0.22 | 0.07 | 0.75 | 78% | 25% | 94% |
| 9 | 0.25 | 0.08 | 0.79 | 75% | 21% | 92% |
| 10 | 0.29 | 0.10 | 0.84 | 72% | 16% | 90% |
| 11 | 0.32 | 0.12 | 0.89 | 68% | 11% | 88% |
| 12 | 0.37 | 0.14 | 0.94 | 63% | 6% | 86% |
| 13 | 0.41 | 0.17 | 1.01 | 59% | -1% | 83% |
| 14 | 0.47 | 0.20 | 1.09 | 53% | -9% | 80% |
| 15 | 0.53 | 0.24 | 1.19 | 47% | -19% | 76% |
| 16 | 0.60 | 0.28 | 1.31 | 40% | -31% | 72% |
| 17 | 0.68 | 0.32 | 1.46 | 32% | -46% | 68% |
| 18 | 0.78 | 0.37 | 1.64 | 23% | -64% | 63% |
| 19 | 0.88 | 0.41 | 1.87 | 12% | -87% | 59% |
| 20 | 1.00 | 0.46 | 2.16 | 0% | -116% | 54% |
| 21 | 1.13 | 0.51 | 2.52 | -13% | -152% | 50% |
| 22 | 1.28 | 0.55 | 2.98 | -28% | -198% | 45% |
| 23 | 1.45 | 0.59 | 3.54 | -45% | -254% | 41% |
| 24 | 1.65 | 0.64 | 4.25 | -65% | -325% | 36% |
